# The association between macrovascular complications and intensive care admission, invasive mechanical ventilation, and mortality in people with diabetes hospitalized for coronavirus disease-2019 (COVID-19)

**DOI:** 10.1186/s12933-022-01657-8

**Published:** 2022-10-19

**Authors:** Gemma Llauradó, Bogdan Vlacho, Matthieu Wargny, Yue Ruan, Josep Franch-Nadal, Pere Domingo, Pierre Gourdy, Pierre-Jean Saulnier, Samy Hadjadj, Sarah H. Wild, Rustam Rea, Bertrand Cariou, Kamlesh Khunti, Dídac Mauricio, for the CORONADO, the ABCD COVID-19 diabetes national audit, HM Hospitales investigators and the Hospital del Mar - Hospital de la Santa Creu i Sant Pau Diabetes Research Group, Juan Antonio Arroyo, Juan-José Chillarón, Rosa Corcoy, Roberto Güerri, Manel Mata-Cases, Emilio Ortega, Matthieu Pichelin, Maëva Saignes, Jeanne Saunier, Selma El Andaloussi, Joëlle Martin-Gauthier, Emily Rebouilleau, Tanguy Roman, Pascale Mahot, Matthieu Pichelin, Anne-Laure Fournier-Guilloux, Nicolas Mauduit, Edith Bigot- Corbel, Anne-Sophie Boureau, Laure De Dekcer, Audrey Ernould, Claire Primot, Anne Seguin, Marielle Joliveau, Sonia Pouvreau, Chloé Fournier, Jeremy Thureau, Edith Fonteneau, Pamela Hublain, Carole Agasse, Mathilde De Kergaradec, Vincent Minville, Fanny Vardon-Bounes, Guillaume Martin-Blondel, Blandine Tramunt, Marie-Christine Turnin, Hélène Hanaire, Jean-Michel Mansuy, Didier Fabre, Marie-Blanche Arhainx, Laurent Cazals, Laure Combes, Emmanuelle Lami, Bruno Megarbane, Pierre Leroy, Jean-François Gautier, Tiphaine Vidal-Trecan, Jean-Pierre Riveline, Jean-Louis Laplanche, Stéphane Mouly, Louis Potier, Ronan Roussel, Malak Taher, Yawa Abouleka, Fetta Yaker, Aurelie Carlier, Anne Boutten, Marilyne Hallot-Feron, Fadila Mourah, Charles Thivolet, Emilie Blond, Muriel Rolland, Josep Verdecho Mendez, Marine Alexandre, Julien Pottecher, Emilie Richer, Laurent Meyer, Florina Luca, Jean-Marc Lessinger, Thibault Bahougne, Bruno Guerci, Siham Benzirar, Catherine Malaplate, Thierry Matton, Julien Poissy, Karine Faure, Pierre Fontaine, Florence Baudoux, Anne Vambergue, Jean David Pekar, Marc Lambert, Cécile Yelnik, Amélie Bruandet, Laurent Petit, Didier Neau, Vincent Rigalleau, Annie Berard, Amandine Galioot, Remy Coudroy, Arnaud Thille, René Robert, France Roblot-Cazenave, Blandine Rammaert, Pierre Jean Saulnier, Xavier Piguel, Nesrine Benhenda, Camille Husson, Celine Olivier, Florence Torremocha, Mathilde Fraty, Marie Flamen D’assigny, Aurelie Miot, Valentin Bossard, Kada Klouche, Alain Makinson, Ariane Sultan, Jean-Baptiste Bonnet, Vincent Foulongne, Florence Galtier, Cécile Aubron, Séverine Ansart, Véronique Kerlan, Pascale Quiniou, Jean- Luc Carre, Stéphane Quesnot, Bruno Laviolle, Carole Schwebel, Olivier Epaulard, Pierre-Yves Benhamou, Cécile Betry, Anne-Laure Borel, Sandrine Lablanche, Dorra Guergour, Catherine Duclos, Emmanuel Cosson, Erwan Guyot, Aurore Deniau, Phucthutrang Nguyen, Yves Reznik, Michael Joubert, Stéphane Allouche, Lydia Guittet, Steven Grange, Manuel Etienne, Gaëtan Prévost, Valéry Brunel, Jean-Christophe Lagier, Didier Raoult, Anne Dutour, Bénédicte Gaborit, Sandrine Boulllu, Patrice Darmon, Adèle Lasbleiz, Mathieu Cerino, Fanny Romain, Marie Houssays, Jean Pierre Quenot, Lionel Piroth, Bruno Vergès, Laurence Duvillard, Bernard Bonnotte, Alain Mercat, Vincent Dubee, Ingrid Allix, Patrice Rodien, Robin Dhersin, Maylis Lebeault, Wojciech Trzepizur, Jocelyne Loison, Antoine Brangier, Pierre Asfar, Pascal Reynier, Françoise Larcher, Françoise Joubaud, Marie-Rita Andreu, Geoffrey Urbanski, Laurent Hubert, Cedric Annweiler, Jean Dellamonica, Johan Courjon, Nicolas Chevalier, Giulia Chinetti, Magda Chafai, Bruno Mourvillier, Firouze Bani-Sadr, Sarra Barraud, Brigitte Delemer, Philippe Gillery, Pascale Labedade, Amélie Chabrol, Alfred Penfornis, Catherine Petit, Coralie Amadou, Maxime Adler, Clément Dubost, Pierre-Louis Conan, Lyse Bordier, Franck Ceppa, Cyril Garcia, Mathilde Sollier, Olivier Dupuy, Sophie Laplance, Olivier Billuart, Marie Joseph Aroulanda, Frédérique Olivier, Florence Ayon, Nathalie Wilhelm, Loic Epelboin, Nadia Sabbah, Aurelie Charpin, Pierre Squara, Olivier Belliard, Claude Dubois, Michel Marre, Johann Auchabie, Roxane Courtois, Thierry Duriez, Tiphaine Mergey, Laura Vallee, Laetitia Seguin, Abdallah Al-Salameh, Jean-Philippe Lanoix, Sandrine Soriot-Thomas, Anne-Marie Bourgeois-Descouls, Rachel Desailloud, Natacha Germain, Bogdan Galusca, Gwenaelle Belleton, Nesrine Marouani, Delia Palaghiu, Amira Hammour, Fernando Berdaguer, Thimothée Klopfenstein, Hajer Zayet, Patrice Winiszewski, Marie Zanusso, Pauline Garnier, Ingrid Julier, Karim Hamzaoui, Sophie Marty-Gres, Tarik El Sadki, Lucile Cadot, Jean-Louis Dubost, Céline Gonfroy, Catherine Campinos, Pascale Martres, Marie Pierre Coulhon, Nicolas Allou, Marwa Bachir, Stella Hoang, Candice Kembellec, Olivia Suply, Fatima Kharcha, Anne-Claire Devouge, Anna Flaus-Furmanuk, Isabelle Madeline, Vincent Ehinger, Sophie Bastard, Loic Raffray, Frederic Renou, Aude Bojarsk, Karine Borsu, Angelique Gorlin, Servane Di Bernardo, Carole Truong Van Ut, Stephane Renaud, Antoine Vignoles, Emilie Foch, Laurie Masse, Hubert Grand, Helene Ferrand, Christelle Raffaitin-Cardin, Hadjer Zellagui, Celine Castang-Brachet, Frederique Boury, Ana Alvarez Tena, Isabelle Moura, Pierre Kalfon, Louis Pasteur, Juliana Darasteanu, Louis Pasteur, Arnaud Monier, Louis Pasteur, Pascal Foucault, Louis Pasteur, Alexandra Depuille, Louis Pasteur, Stéphanie Laugier-Robiolle, Patrick Caneiro, Maud Basso, Etienne Larger, Samir Bouam, Wahiba Benzenati, Leila Ait Bachir, Camille Cussac Pillegand, Marc Vasse, Christophe Michard, Nathanaëlle Montanier, Luc Millot, Françoise Crepet, Danielle Ratsimba, Kevin Bouiller, Sophie Borot, Isabelle Bruckert, Annie Clergeot, Franck Schillo, Dorothée Vignes, Muriel Bourgeon-GhittoriLachgar, Claire Lambert De Cursay, Stéphane Levante, Jean Charles Auregan, Antoine Merlet, Cécile Zaragoza, Gwénaëlle Arnault, Anne-Gaëlle Le Loupp, Olivier Lesieur, Mariam Roncato-Saberan, Didier Gouet, Romain Lemari, Hong-An Allano, Emmanuel Vivier, Caroline Pariset, Cédric Luyton, Lucien Marchand, Fanny Doroszewski, Matthieu Pecquet, Laurent Perard, Sylvie Vuillermoz-Blas, Nicolas Kacki, Patricia Charrier, Amélie Ducet-Boiffard, Françoise Desroys Du Roure, Olivier Bourron, Dominique Bonnefont-Rousselot, Suzanne Laroche, Franck Phan, Agnès Hartemann, Cyrielle Caussy, Emmanuel Disse, Emilie Blond, Claude Guerin, Thomas Perpoint, Philippe Moulin, Régine Cartier, Geoffroy Hariri, Dorothée Chopin, Camille Vatier, Nathalie Bourcigaux, Emmanuelle Chaigneau, Sophie Christin-Maitre, Bruno Donadille, Bruno Feve, Sophie Lamothe, Julie Sarfati, Pascal Pernet, Anne Chambon, Delphine Demarsy, Hugo Campagne, Françoise Latil-Plat, Monica Berne, Marilyne Grinand, Marion Touzet, Aydrey Zabulon, Jocelyne Craspag, Catherine Ledoux, Cedric Contaret, Blandine Janand-Delenne, Anaïs Giraud, Marie Lou Lacrimini, Joëlle Arrivie, Deborah Ancelle, Carine Guillois, Bénédicte Fremy, Amina Chaalal, Gaëlle Barrande, Anne Dorange, Eglantine Rouanet, Dominique Seret-Begue, Audrey Saoud, Anne-Marie Guedj, Nathalie Bedos, Fritz-Line Velayoudom, Marie Dumas, Benoite Gonda, Christine Coffin, Stéphanie Gibiat, Myriam Lungo, Chantal Bully, Pierre Serusclat, Stella Bully, Patricia Carre, Jean-Philippe Leberre, Carlos Elkhoury, Marine Thieux, Laetitia Paradisi-Prieur, Emma Wilmot, Sarah Wild, Ben Field, Parth Narendran, Rajiv Gandhi, Sophie Harris, Dinesh Nagi, Robert Ryder, Jim Davies, Steve Harris, Oliver Freeman, Ben Maylor, Kinga A. Várnai, Gail Roadknight, Melissa Cul, Amy Edwards, Susan Gelding, Kirun Gunganah, Pyei Aung, Moulinath Banerjee, Ali Chakera, Dominique Rouse, Syed Haris Ahmed, Ho Yee Cheung, Hywel Roberts, Susan Seal, Syed Saah Shah, Amir Hayat, Cynthia Mohandas, Htet Htet Aung, Su Khant Chel, Nyan Lin, Kavitia Narula, Furruq Quadri, Su Lei Yin, Yin Yin, Alamin Alkundi, Abdelmajid Musa, Emma Birbeck, Charles Bodmer, Irene Bossman, Sathis Kumar, Umesh Dashora, Elizabeth Toubi, Mansoor Zafar, Vinod Patel, Amitha Gopinath, Belinda Allan, Remat Karim, Dharshana Appuhamillage, Khubaib Ayoub, Sophie Harris, Charmaine Ilangaratne, Maliha Iqbal, Rory Maclean, Omar Mustafa, Susan Baxter, Malgorzata Adamus, Kevin Baynes, Siva Sivappriyan, Ryan D’Costa, Dinesh Nagi, Vernon Parfitt, Mazharul Islam, Sadia Nasir, Gail Roadknight, Kinga Várnai, Senthil Vasan, Vilashini Arul Devah, Foteini Kavvoura, Lina Ficken, James Gilham, Vincent Simpson, Neil Walker, Umaira Aziz, Efthimia Karra, Dipesh Patel, Miranda Rosenthal, Tracy Curran, Angela Paisley, Melissa Cull, Parijat De P, May Thin Khine, Pari Qayyam, Robert Ryder, Priscilla Sarkar, Rajiv Gandhi, Ben Field, James Clark, Vesna Hogan, Lauren Jackson, Jamie-Leigh Williamson, R. Younes, Lucy Robin, Lydia Grixti, Suann Tee, Abilash Sathya, Emma Wilmot, Mayank Patel, Catherine Holmes, Wasim Hanif, Sandip Ghosh, Parth Narendran, Ehtasham Ahmad, Ejaz Ahmed, Melanie Davies, Steven Hartshorn, Kamlesh Khunti, Lee Simons, David Webb, Ben Maylor, Jim Davies, Oliver Freeman, Steve Harris, Anupam Brahma, Seshadri Pramodh, Katy Frew, Alison Mackenzie, Abigail Wild, Helen Casey, Deborah Morrison, Conor McKeag, Anne Sillars, Angus Stirling, Fiona Smeeton, Syed Muhammad, Kofi Obuobie, Win Yin, Neera Agarwal, Mike Atkinson, Sai Ambati, Rahim Khan, Preethi Nalla, Arshiya Tabasum, Stamatios Zouras, Akhila Mallipedhi, Richard Chudleigh, David Williams, Mallory Cianferani, Lisa Ludwig, Caroline Paul, Hamoud Lachgar

**Affiliations:** 1 Department of Endocrinology and Nutrition, Hospital del Mar, Institut Hospital del Mar d’Investigacions Mèdiques (IMIM), Barcelona, Spain; 2 Department of Endocrinology and Nutrition, Center for Biomedical Research on Diabetes and Associated Metabolic Diseases (CIBERDEM), Instituto de Salud Carlos III (ISCIII), Barcelona, Spain; 3 DAP_CAT Group, Unitat de Suport a la Recerca Barcelona, Fundació Institut Universitari Per a la Recerca a l’Atenció Primària de Salut Jordi Gol i Gorina (IDIAPJGol), Barcelona, Spain; 4 Institut de Recerca Hospital de la Santa Creu i Sant Pau, Barcelona, Spain; 5 L’Institut du Thorax, Université de Nantes, CHU Nantes, CNRS, Nantes, Inserm France; 6 CHU de Nantes, CIC Inserm 1413, Clinique Des Données, Nantes, France; 7 Oxford Centre for Diabetes, Endocrinology and Metabolism, Oxford University Hospitals NHS Foundation Trust, Oxford, UK; 8 Oxford NIHR Biomedical Research Centre, Oxford, UK; 9 Primary Health Care Center Raval Sud, Gerència d’Àmbit d’Atenció Primaria, Institut Català de la Salut, Barcelona, Spain; 10 Infectious Diseases, Hospital de la Santa Creu i Sant Pau and Sant Pau Biomedical Research Institute (IIB Sant Pau), Barcelona, Spain; 11 CHU de Toulouse & UMR1297/I2MC, Inserm, Université de Toulouse, Toulouse, France; 12 Centre d’Investigation Clinique CIC 1402, Université de Poitiers, Inserm, CHU de Poitiers, Poitiers, France; 13 Usher Institute, University of Edinburgh, Edinburgh, UK; 14 Diabetes Research Centre, Leicester General Hospital, University Hospitals of Leicester NHS Trust, Leicester, UK; 15 Department of Endocrinology and Nutrition, Hospital de la Santa Creu i Sant Pau and Sant Pau Biomedical Research Institute (IIB Sant Pau), Barcelona, Spain

**Keywords:** Diabetes, Macrovascular disease, Mortality, COVID-19

## Abstract

**Background:**

It is not clear whether pre-existing macrovascular complications (ischemic heart disease, stroke or peripheral artery disease) are associated with health outcomes in people with diabetes mellitus hospitalized for COVID-19.

**Methods:**

We conducted cohort studies of adults with pre-existing diabetes hospitalized for COVID-19 infection in the UK, France, and Spain during the early phase of the pandemic (between March 2020—October 2020). Logistic regression models adjusted for demographic factors and other comorbidities were used to determine associations between previous macrovascular disease and relevant clinical outcomes: mortality, intensive care unit (ICU) admission and use of invasive mechanical ventilation (IMV) during the hospitalization. Output from individual logistic regression models for each cohort was combined in a meta-analysis.

**Results:**

Complete data were available for 4,106 (60.4%) individuals. Of these, 1,652 (40.2%) had any prior macrovascular disease of whom 28.5% of patients died. Mortality was higher for people with compared to those without previous macrovascular disease (37.7% vs 22.4%). The combined crude odds ratio (OR) for previous macrovascular disease and mortality for all four cohorts was 2.12 (95% CI 1.83–2.45 with an I^2^ of 60%, reduced after adjustments for age, sex, type of diabetes, hypertension, microvascular disease, ethnicity, and BMI to adjusted OR 1.53 [95% CI 1.29–1.81]) for the three cohorts. Further analysis revealed that ischemic heart disease and cerebrovascular disease were the main contributors of adverse outcomes. However, proportions of people admitted to ICU (adjOR 0.48 [95% CI 0.31–0.75], I^2^ 60%) and the use of IMV during hospitalization (adjOR 0.52 [95% CI 0.40–0.68], I^2^ 37%) were significantly lower for people with previous macrovascular disease.

**Conclusions:**

This large multinational study of people with diabetes mellitus hospitalized for COVID-19 demonstrates that previous macrovascular disease is associated with higher mortality and lower proportions admitted to ICU and treated with IMV during hospitalization suggesting selective admission criteria. Our findings highlight the importance correctly assess the prognosis and intensive monitoring in this high-risk group of patients and emphasize the need to design specific public health programs aimed to prevent SARS-CoV-2 infection in this subgroup.

**Supplementary Information:**

The online version contains supplementary material available at 10.1186/s12933-022-01657-8.

## Background

Coronavirus disease 2019 (COVID-19) is caused by the severe acute respiratory syndrome coronavirus 2 (SARS-CoV-2) and is characterized by a variable clinical presentation that ranges from asymptomatic infection to fatal multi-organ damage and mortality [1, 2]. Since the emergence of SARS-CoV-2 in December 2019, cases of COVID-19 have rapidly increased worldwide. The updated WHO estimates on August 19th, 2022, reported 590,659,276 confirmed cases, including 6,440,163 deaths worldwide (https://covid19.who.int). The case fatality for COVID-19 has been estimated to be 0.5–1.0% [3, 4]. Nevertheless, certain characteristics, including increasing age, male sex, ethnicity, socio-economic deprivation, and comorbidities, have been associated with a higher risk of severe COVID-19 or death [5–7].

COVID-19 pandemic has had a large negative impact on both diabetes management [8] and diabetes-related mortality [9]. As well, pre-existing diabetes mellitus has been considered a risk factor for increased COVID-19 severity and worse outcomes, including higher mortality, irrespective of age and comorbidity status [7]. The estimates of diabetes prevalence in those who have died of COVID-19 range from 20 to 30% [10, 11]. A recent meta-analysis showed that people with diabetes were at higher risk of COVID-19-related mortality in comparison to people without diabetes [11]. In addition, diabetes is associated with more than double the risk for ICU admission and more than triple the risk of death compared to people without diabetes [12]. Therefore, identifying which clinical factors are associated with greater morbidity and mortality would be useful for the prevention and management of high-risk groups during future waves of the pandemic. In that sense, few studies have examined the possibility that micro- and macrovascular complications contribute to susceptibility to acute organ injury [13, 14] but with contradictory results [15].

Our study aimed to assess whether the presence of macrovascular complications (ischemic heart disease, stroke, or peripheral artery disease) prior to hospital admission is associated with intensive care unit admission, mechanical ventilation, and mortality in people with diabetes mellitus hospitalized for COVID-19 in four European cohorts.

## Methods

### Study design and participants

Retrospective data from hospitalized adults with pre-existing diabetes and concomitant COVID-19 infection were collected in the UK, France, and Spain. Adults with hyperglycaemia at admission but not pre-existing or subsequent diagnosis of diabetes (based on WHO criteria) were excluded from the analysis [16]. COVID-19 was defined as a SARS-CoV-2 infection confirmed by quantitative PCR (qPCR) performed on nasopharyngeal samples obtained by trained personnel and/or by fulfilling clinical and radiological diagnostic criteria at hospital admission. Further descriptions of each dataset have been published previously [17].

### United Kingdom: association of British clinical diabetologists (ABCD) COVID-19 audit

The NHS supports audits with clear guidance for the contributing centers on using routine clinical practice data submitted anonymously via the secure NHS network [18]. Clinicians participating in the ABCD COVID-19 audit submitted data for adults with pre-existing type 1 and type 2 diabetes admitted with COVID-19 from hospitals across the UK. The audit is registered with Oxford University Hospitals NHS Foundation Trust (OUH), a Data Protection Impact Assessment was carried out and the audit was approved by the OUH Caldicott Guardian and the Public Benefit and Privacy Panel in Scotland (reference 2021-0111).

### France: CORONADO (CORONAvirus-SARS-CoV-2 and diabetes outcomes)

The CORONADO study described the phenotypic characteristics and prognosis of people with diabetes admitted with COVID-19 between March 10 and April 10, 2020 [13, 19]. CORONADO is a cohort study from French hospitals volunteering to share data on hospitalized COVID-19 patients with diabetes. The study was sponsored by the Nantes University Hospital and designed in accordance with the Declaration of Helsinki. It obtained all regulatory approvals.

### Spain—HM Hospitales cohort

The six hospitals in the HM Hospitales group collected anonymized observational data for people infected with COVID-19 during the first wave of the pandemics. This dataset is made available to researchers via “Covid Data Save Lives” [20]. The electronic hospital health records were collected for admitted persons, including pre-existing disease status, medication use, demographic, and outcome. A subset of people with pre-existing diabetes from this cross-sectional database was used in this study. Before access was granted, a formal petition, specific study protocol, and ethics committee approval were obtained. The study was approved by the Ethics Committee of the Primary Health Care University Research Institute (IDIAP) Jordi Gol, Barcelona (approval number: 20/089-PCV).

### Spain—Barcelona cohort

An observational cohort study was conducted at the Hospital del Mar and Hospital de la Santa Creu i Sant Pau, two tertiary hospitals in Barcelona providing healthcare to 800,000 people. The two hospitals from Barcelona (Catalonia) collected anonymized observational data for people infected with COVID-19 during the first wave. Demographic, clinical, epidemiological, and whole-episode (laboratory workup, vital signs, treatment) data were extracted from electronic medical records using a standardized data collection method. All patients with type 2 diabetes mellitus admitted for COVID-19 between March and April 2020 were included. The Hospital del Mar Institutional Ethics Committee (CEIm-2020/9352) and the Hospital de la Santa Creu I Sant Pau Ethics Committee (HSCSP-20/117) approved the study and waived the informed consent need due to the study’s nature.

### Data collection: definitions and outcomes

Demographic data included: age, sex, and type of diabetes. UK and France collected ethnicity data (White/Europid, Black/African, Asian/Asian, Other/Middle East and North African (MENA)). Medication use at the point of admission was collected with particular focus on those medications associated with diabetes or diabetes-related comorbidities. Microvascular disease (including retinopathy, neuropathy, and nephropathy) was collected for the UK, French and Spanish (Barcelona cohort) cohorts. The Spanish cohort (HM Hospitales) collected data on the presence of chronic kidney disease (CKD) alone based on clinical coding records. CKD was defined by eGFR < 60 ml/min or the presence of macroalbuminuria (urinary albumin-to-creatinine ratio ≥ 300 mg/g) [21].

### Definition of macrovascular complications

**Fig. 1:**
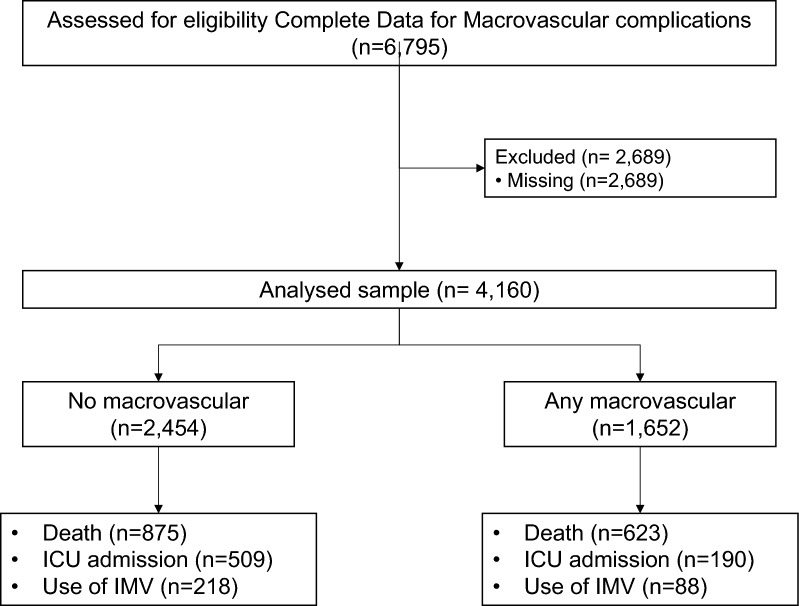
Inclusion and exclusion criteria

History of macrovascular disease was collected for all datasets. The presence of macrovascular complications was defined according to the presence of a previous history of ischemic heart disease (including a history of myocardial infarction and/or coronary artery revascularization or heart failure), cerebrovascular disease (including history of stroke or transient ischemic attack – TIA-) and/or peripheral artery disease (amputation owing to ischemic disease and/or lower limb artery revascularization). Data were obtained based on the information recorded in medical records or according international ICD10 classification. Of the 6,795 people included, 4,106 had complete data for macrovascular complications (all four cohorts) and/or the rest of the variables included (French, UK and Spanish HM Hospitales cohorts). The flowchart of the study is summarized in Fig. 1. The descriptive analysis compared the characteristics of people with and without complete data for macrovascular disease. The comparison of the clinical characteristics of people with complete data compared with those with missing data for macrovascular complications is shown in Table 1.

**Table 1:** Comparison of the clinical characteristics of people with complete vs missing data for macrovascular complications

	UK		FRANCE		Spain—HM		Spain—Barcelona	
	Complete data (n = 1846)	Missing data (n = 1333)	Complete data (n = 1510)	Missing data (n = 1333)	Complete data (n = 406)	Missing data (n = 0)	Complete data (n = 344)	Missing data (n = 23)
Age (mean, SD) years	72.5 (13.9)	70.8 (14.4)	70.1 (13.0)	69.1 (13.3)	74.7 (11.9)	–	71.1 (13.9)	74.0 (14.7)
Missing, n	0	6	0	0	0	–	1	0
Men, n (%)	1140 (62)	802 (60)	933 (62)	882 (66)	250 (62)	–	202 (59)	11 (48)
Missing, n	0	5	0	0	0	–	0	0
BMI (mean, SD) kg/m^2^	29.3 (7.2)	28.6 (6.9)	29.9 (5.7)	28.9 (6.1)	NA	–	29.8 (6.6)	33.2 (5.8)
Missing, n	0	860	0	124	NA	–	295	21
Ethnicity						–		
White, n (%)	1291 (70)	624 (60)	893 (59)	517 (56)	NA	–	77 (87)	18 (86)
Asian, n (%)	275 (15)	167 (16)	55 (4)	33 (4)	NA	–	1 (1)	1 (5)
Black, n (%)	116 (6)	94 (9)	242 (16.0)	179 (20)	NA	–	2 (2)	0 (0)
Other, n (%)	164 (9)	157 (15)	320 (21)	187 (20)	NA	–	9 (10)	2 (10)
Missing, n	0	291	0	417	NA	–	255	2
Type of diabetes								
Type 1, n (%)	93 (5)	62 (5)	48 (3)	13 (1)	4 (1)	–	5 (5)	0 (0.0)
Type 2, n (%)	1753 (95)	1163 (95)	1462 (97)	1079 (99)	402 (99)		88 (95)	23 (100.0)
Missing, n	0	108	0	241	0	–	251	0
Hypertension, n (%)	1286 (70)	774 (66)	1204 (80)	953 (73.0)	286 (70)	–	278 (81)	18 (78)
Missing, n	0	166	0	28	0	–	0	0
Dyslipidaemia, n (%)	NA	NA	767 (51)	521 (42)	198 (49)	–	NA	NA
Missing, n	-	-	0	81	0	–	–	–
Microvascular disease, n (%)	807 (44)	242 (39)	701 (46)	206 (42)	38 (9)	–	15 (17)	0
Missing, n	0	710	0	848	0	–	257	23
Macrovascular disease, n (%)	804 (44)	272 (40)	640 (42)	477 (39)	98 (24)	–	110 (32)	NA
Missing, n	0	652	0	109	0	–	0	23
Death (in-hospital mortality), n (%)	715 (39)	524 (39)	290 (19)	294 (22)	79 (20)	–	88 (28)	8 (36)
Missing, n	0	0	0	7	0	–	18	1
ICU admission, n (%)	193 (10)	125 (13)	412 (27)	439 (33)	35 (9)	–	59 (17)	1 (5)
Missing, n	0	337	0	14	0	–	1	1
Use of IMV, n (%)	NA	NA	269 (18)	297 (23)	37 (9)	–	NA	NA
Missing, n	-	-	0	14	0	–	–	–

*BMI* body mass index, *ICU* intensive care unit, *IMV* Mechanical ventilation, *SD* Standard deviation

### Outcomes

The primary outcome was all-cause mortality (French data were collected to day 28 after admission, and Spanish and UK data included mortality during the whole hospital episode). The secondary outcomes were intensive care unit (ICU) admission for all four cohorts and use of invasive mechanical ventilation (IMV) during the hospitalization for the French and the Spanish HM Hospitales cohorts for which IMV data were available.

### Statistical analysis

All quantitative data were tested for normality. Clinical characteristics were expressed as the number (percentage) of participants for categorical variables, mean ± standard deviation (SD) for normally distributed continuous variables, or median (25–75th percentile) for non-normally distributed continuous variables. Regulatory issues prevented us from sharing and combining individual level data from each contributing country, so, multivariable logistic regression models were used to analyse the association between the presence of macrovascular disease (main exposure) and primary (death) and secondary outcomes (ICU admission and IMV) in each country’s data separately. Logistic regression models were then used to adjust for potential confounders: age and sex (model 1), age, sex, type of diabetes, arterial hypertension, and the presence of microvascular disease (model 2) and age, sex, type of diabetes, arterial hypertension, microvascular disease, ethnicity, and BMI (model 3–data not available for HM cohort). Logistic regressions were performed using R in each contributing country; country-specific odds ratios were then pooled in both common effect and random effect meta-analysis (as needed according to I^2^ statistic) and using the inverse variance method. Heterogeneity across studies was evaluated using the I^2^ statistic. Results were expressed as odds ratio (OR) and 95% confidence interval (95% CI) and p-values < 0.05 were considered statistically significant. Statistical analyses were performed with R statistical software version 3.6.1 (https://www.r-project.org/).

## Results

The UK ABCD COVID-19 audit collected data on 3,179 people with diabetes from over 40 hospitals between March and October 2020. Of these, 1,846 (58.1%) had complete data required for this study and were included in the analysis. CORONADO investigators collected data on 2,843 people with diabetes from 68 hospitals, with 1,510 (53.1%) having complete data. Spanish investigators from the HM Hospitales collected data on 2,310 individuals at six hospitals. There was complete data for 406 individuals (100.0%) with pre-existing diabetes. Finally, the Spanish investigators from Hospital del Mar and Hospital de la Santa Creu i Sant Pau collected data on 367 individuals, with 344 (93.7%) having complete data. A comparison of the complete dataset to that with missing data within all the countries (Table 1). Ethnicity data were not available for the Spain – HM Hospitales cohort.

The baseline characteristics of the cohort of patients in each country are summarized in Table 2. Data related to macrovascular disease status was available for 4,106 people. Of these, 1,652 (40.2%) had any prior macrovascular disease, 1,339 (32.6%) had a previous history of ischemic heart disease, 520 (12.7%) had previous cerebrovascular disease, and 457 (11.1%) had previous peripheral artery disease. In the four included cohorts, people with a history of macrovascular disease were older, had a higher percentage of men and had a higher prevalence of hypertension, dyslipidemia, and microvascular complications than the group with no history of macrovascular disease.

**Table 2:** Clinical characteristics of subjects according to macrovascular status

	UK				FRANCE				Spain—HM				Spain—Barcelona			
	All (n = 1846)	No MACRO (n = 1042)	Any MACRO (n = 804)	P	All (n = 1510)	No MACRO (n = 870)	Any MACRO (n = 640)	P	All (n = 406)	No MACRO (n = 308)	Any MACRO (n = 98)	P	All (n = 344)	No MACRO (n = 234)	Any MACRO (n = 110)	p
Age (mean, SD) years	72.5(13.9)	69.1(14.8)	76.8(11.2)	< 0.001	70.1(13.0)	66.7(13.0)	74.7(11.5)	< 0.001	74.7(11.9)	72.8(11.9)	80.6(9.7)	< 0.001	71.1(13.9)	69.3(13.9)	74.8(13.0)	< 0.001
Men, n (%)	1140(62)	610 (59)	530 (66)	0.001	933 (62)	495(57)	438 (68.4)	< 0.001	250(62)	185(60)	65(66)	0.322	202 (58.9)	129 (55.1)	73 (67.0)	0.038
Ethnicity, n (%)				< 0.001				< 0.001				NA				0.688
White	1291(70)	655(63)	626(78)		893(59)	467(54)	426(66.6)		NA	NA	NA		77(87)	58(84)	19(95)	
Asian	275(15)	200(19)	75 (9)		55(4)	39(5)	16(2.5)		NA	NA	NA		1 (1)	1 (2)	0 (0)	
Black	116(6)	64(6)	52 (6)		242(16)	173(20)	69(10.8)		NA	NA	NA		2 (2)	2 (3)	0 (0)	
Other	164(9)	123(12)	41 (5)		320(21)	191(22)	129(20.2)		NA	NA	NA		9 (10)	8 (12)	1 (5)	
Type of diabetes																
Type 1, n (%)	93(5)	59(6)	34(4)	0.200	48(3)	33(4)	15(2.3)	0.107	4 (1)	3 (1)	1 (1)	1.000	5 (5)	5 (7)	0 (0.0)	0.642
Type 2, n (%)	1753(95)	983(94)	770(96)		1462(97)	837(96)	625(97.7)		402(99)	305(99)	97(99)		88 (95)	68 (93)	20(100.0)	
Oral agents, n (%)	1123(61)	673(65)	450(56)	< 0.001	1092(72)	684(79)	408(64)	< 0.001	83 (20)	68 (22)	15(15)	0.192	274 (80)	189 (81)	85 (78)	0.452
Insulin,n (%)	696(38)	402(39)	294(37)	0.40	678(44.9)	320(36.8)	358(56)	< 0.001	79 (20)	61 (20)	18 (18)	0.868	97 (28)	55 (24)	42 (39)	0.004
Hypertension, n (%)	1286(70)	713(68)	573(71)		1204(80)	635(73)	569(88.9)	< 0.001	286(70)	207(67)	79(81)	0.016	278(81)	176(75)	102(93)	< 0.001
RAASi, n (%)	1319(71)	715(69)	604(75)	0.003	858(56.9)	446(51.3)	412(64.5)	< 0.001	153 (38)	115 (37)	38 (39)	0.892	199 (58)	131 (56)	68 (62)	0.194
Dyslipidaemia, n (%)	NA	NA	NA	NA	767(51)	363(42)	404(63.1)	< 0.001	198(49)	141(46)	57(58)	0.043	NA	NA	NA	NA
Statins, n (%)	1158(63)	625(60)	533(66)	0.006	764(50.6)	353(40.6)	411(64.3)	< 0.001	107 (26)	60 820)	47 (48)	< 0.001	193 (56)	112 (48)	81 (74)	< 0.001
BMI	29.3 (7.2)	29.9 (7.2)	28.6 (7.1)	< 0.001	29.5 (5.9)	29.9 (5.7)	28.9 (6.1)	0.02	NA	NA	NA	NA	29.8 (6.7)	29.3 (6.4)	33.3 (8.1)	0.176
Diabetes duration	NA	NA	NA	NA	12 [6; 20]	10 [5; 18]	15 [10; 23]	< 0.001	NA	NA	NA	NA	NA	NA	NA	NA
HbA1c (%), mean (SD)	7.8 (4.2)	7.9 (4.3)	7.8 (4.1)	0.270	8.1 (1.8)	8.3 (2.0)	7.9 (1.7)	< 0.001	8.1 (1.7)	8.3 (1.8)	7.7 (1.4)	0.230	7.5 (1.5)	7.3 (1.4)	8.3 (1.8)	0.022
HbA1c mmol/mol, mean (SD)	62 (22)	63 (23)	62 (21)	0.270	65.2(20.2)	67.1(21.5)	62.7(18.2)	< 0.001	65.0(18.2)	67.2(20.2)	60.7(15.4)	0.230	58.9(16.9)	56.6(15.4)	67.2(19.7)	0.022
Microvascular disease, n (%)																
Diabetic retinopathy, n (%)	449(24)	216(22)	233(30)	< 0.001	252(17)	104(12)	148(23)	< 0.001	1(0.2)	1(0.3)	0(0.0)	1.000	8/87 (9)	7/71 (10)	1/16 (6)	0.652
Diabetic kidney disease, n (%)	420(23)	156(15)	264(33)	< 0.001	569 (38)	220(25)	349(54.5)	< 0.001	31(7.6)	12(4)	19(19)	< 0.001	6/87 (7)	5/71 (7)	1/16 (6)	0.910
Macrovascular disease, n (%)																
Ischemic heart disease/Heart failure, n (%)	560(30)	0 (0.0)	560(70)	–	410(27)/185(13)	0(0)	410(64)/185(39)	–	54(13.3)/34(8.4)	0 (0.0)	54(55)/34(35)	–	96 (28)	0 (0.0)	96 (88)	–
TIA/Stroke, n (%)	295(16)	0 (0.0)	295(37)	–	188(13)	0(0)	188(29.4)	–	25 (6.2)	0 (0.0)	25(26)	–	12 (3.5)	0 (0.0)	12 (11)	–
Peripheral artery disease, n (%)	246(13)	0 (0.0)	246(31)	–	198(13)	0(0)	198(31.0)	–	10 (2.5)	0 (0.0)	10(10)	–	3 (1)	0 (0.0)	3 (2.7)	–
CKD, n (%)	NA	NA	NA	NA	NA	NA	NA	NA	51 (12.6)	22(7)	29(30)	< 0.001	NA	NA	NA	NA
Death (in-hospital mortality), n (%)	715(39)	326(31)	389(48)	< 0.001	290(19)	118(14)	172(26.9)	< 0.001	79(19.5)	59(19)	20(20)	0.900	88(25.6)	46(19.7)	42(38.3)	< 0.001
ICU admission, n (%)	193(10)	155(15)	38(5)	< 0.001	412(27)	282(32)	130(20.3)	< 0.001	35(8.6)	28(9)	7 (7)	0.695	59(17.2)	44(18.8)	15(13.6)	0.249
Use of IMV, n (%)	NA	NA	NA	NA	269 (18)	189(22)	80 (12.5)	< 0.001	37 (9.1)	29 (9)	8 (8)	0.862	NA	NA	NA	NA

**Fig. 2:**
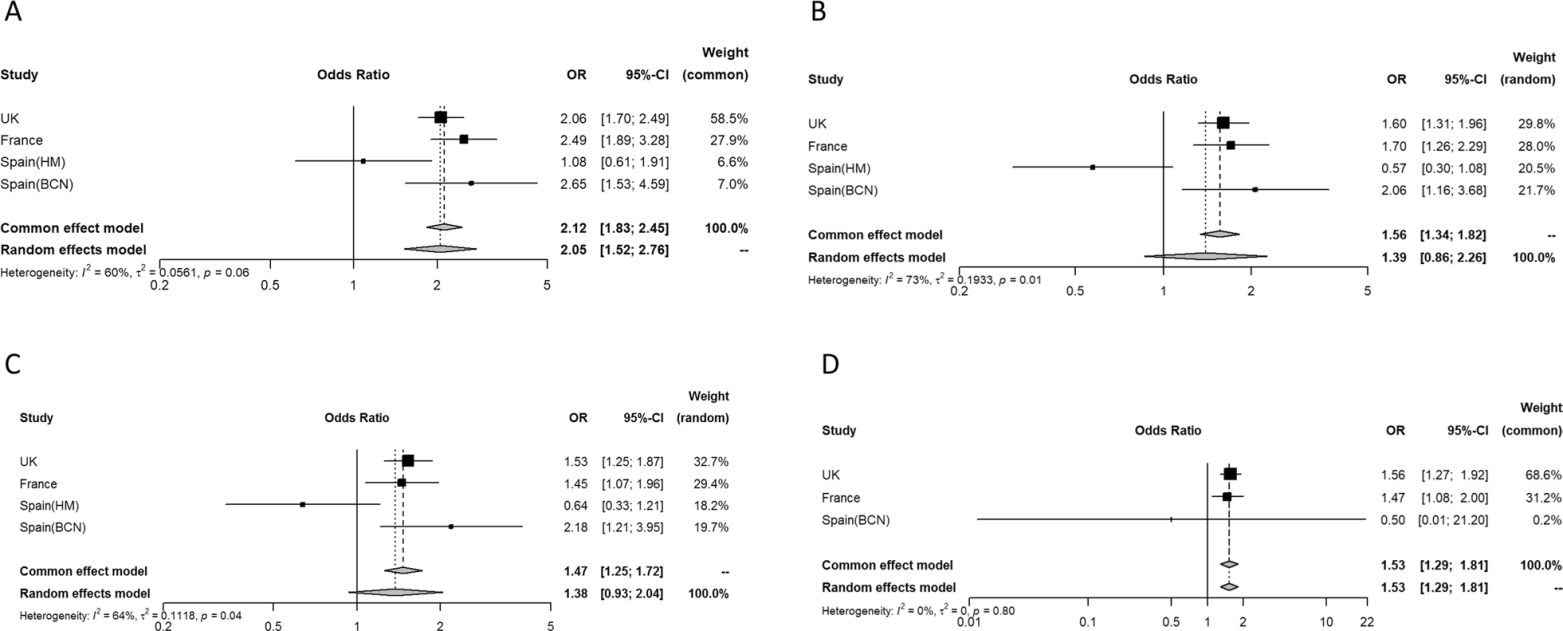
Odds ratio for the association between mortality and the presence of previous macrovascular disease in each of the four cohorts and overall (**A**). I^2^ indicates heterogeneity in the estimates. Odds ratio estimates adjusted for: model 1: age and sex (**B**); model 2: model 1 + type of diabetes, arterial hypertension, and the presence of microvascular disease (**C**); model 3: model 2 + ethnicity and BMI (**D**). HM cohort is excluded from model 3 due to lack of ethnicity data

*BMI* body mass index, *CKD* chronic kidney disease, *ICU* intensive care unit, *IMV* Mechanical ventilation, *SD* Standard deviation,

In total, 1,172 (28.5%, range 19.2–39.0%) people died. Mortality was higher for people with compared to without previous macrovascular disease (37.7% vs 22.4%). The combined crude odds ratio (OR) for previous macrovascular disease and mortality was 2.12 (95% CI 1.83–2.45) (Fig. 2A), with moderate heterogeneity (I^2^ 60%). In the multivariable analyses, the results were attenuated after adjusting for age and sex (model 1: OR 1.39 [95% CI 0.86–2.26]) (Fig. 2B) and age, sex, type of diabetes, arterial hypertension, and the presence of microvascular disease (model 2: OR 1.38 [95% CI 0.93–2.04]) (Fig. 2C). The final model and additional adjustment for ethnicity and BMI (in a subset including 3 of the 4 cohorts) showed similar results (OR 1.53 [95% CI 1.29–1.81]) (Fig. 2D). Further, each component of macrovascular complications was analysed separately, to know which of the 3 diseases contributed the most. Both ischemic heart disease (unadjusted OR 1.78 [95% CI 1.20–2.63]), cerebrovascular disease (unadjusted OR 1.91 [95% CI 1.57–2.34]) and peripheral artery disease (unadjusted OR 1.70 [95% CI 2.38–2.10]) were associated with higher mortality (Additional file 1: Fig. S1, Additional file 2: Fig. S2, Additional file 3: Fig. S3). The results were maintained significant after further adjustments except for peripheral artery disease (Additional file 1: Fig. S1, Additional file 2: Fig. S2, Additional file 3: Fig. S3).

**Fig. 3:**
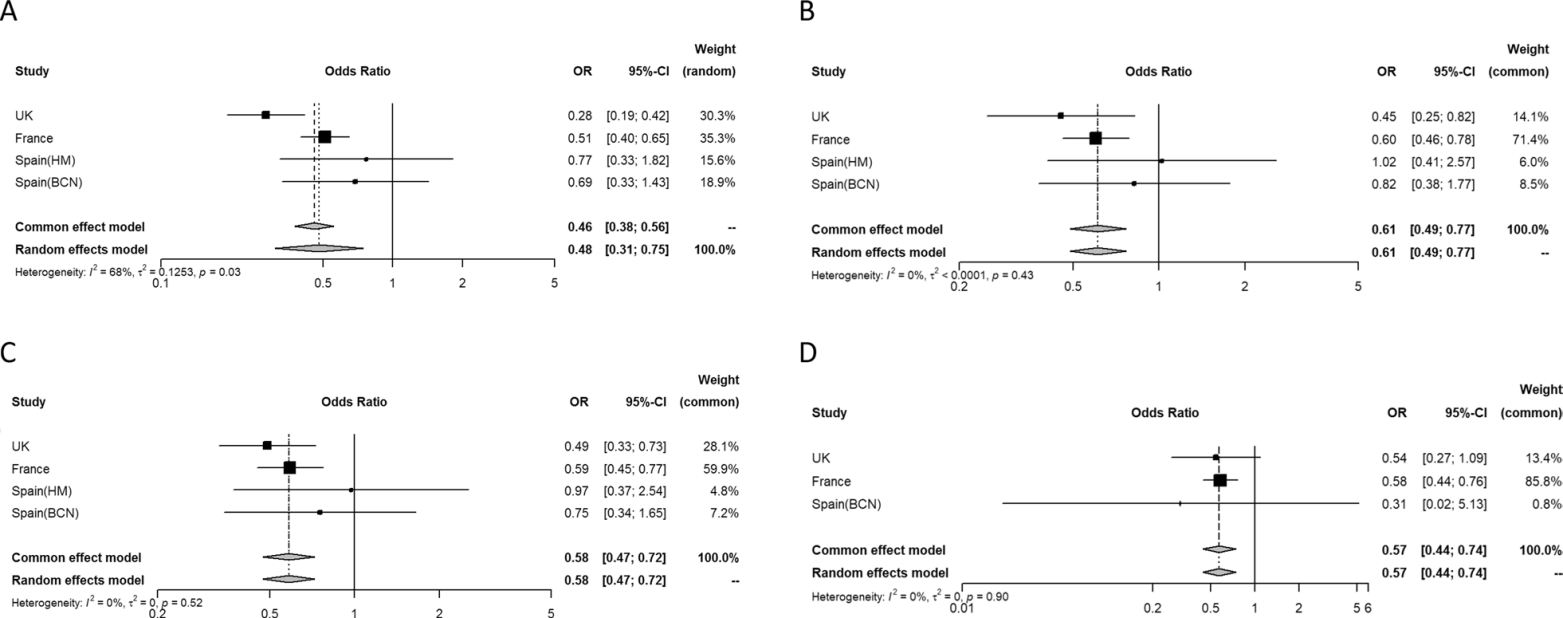
Odds ratio for the association between intensive care unit admission and the presence of previous macrovascular disease in each of the four cohorts and overall (**A**). I^2^ indicates heterogeneity in the estimates. Odds ratio estimates adjusted for: model 1: age and sex (**B**); model 2: model 1 + type of diabetes, arterial hypertension, and the presence of microvascular disease (**C**); model 3: model 2 + ethnicity and BMI (**D**). HM cohort is excluded from model 3 due to lack of ethnicity data

**Fig. 4:**
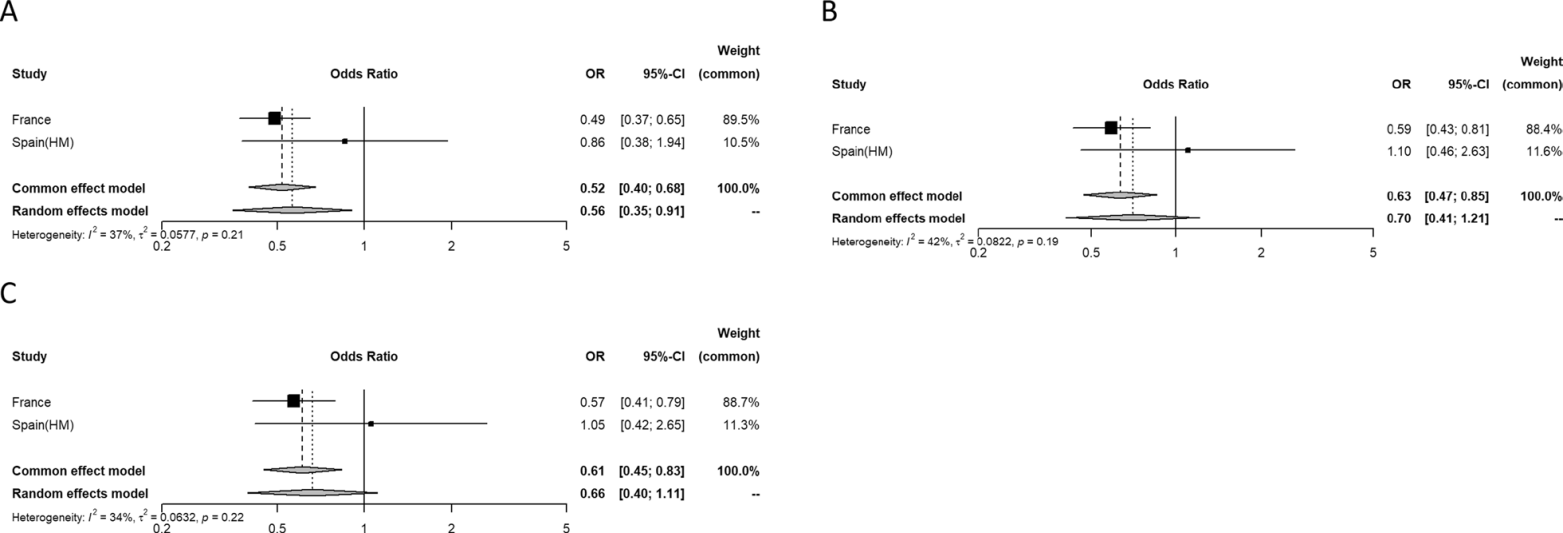
Odds ratio for the association between use of invasive mechanical ventilation during the hospitalization and the presence previous of macrovascular disease in each of the two cohorts and overall (**A**). I^2^ indicates heterogeneity in the estimates. Odds ratio estimates adjusted for: model 1: age and sex (**B**); model 2: model 1 + type of diabetes, arterial hypertension, and the presence of microvascular disease (**C**)

Regarding the secondary outcomes, 699 people (15.7%, range 8.6–22.3%) were admitted to ICU, and 306 (13.6%, range 9.1–17.8%) required IMV during hospitalization. The proportions admitted to ICU or treated with IMV during hospitalization were lower for those patients with previous macrovascular disease (11.5% vs 20.7% and 11.9% vs 18.5%, respectively). The overall odds ratio (OR) for previous macrovascular disease and ICU admission was 0.48 (95% CI 0.31–0.75) in the unadjusted analyses (Fig. 3A), with moderate heterogeneity (I^2^ 68%). In the multivariable analyses, the estimates were similar after adjusting for age and sex (model 1: OR 0.61 [95% CI 0.49–0.77]) (Fig. 3B); age, sex, type of diabetes, arterial hypertension, and the presence of microvascular disease (model 2: OR 0.58 [95% CI 0.47–0.72]) (Fig. 3C); and age, sex, type of diabetes, arterial hypertension, microvascular disease, ethnicity, and BMI (model 3: OR 0.57 [95% CI 0.44–0.74]) (Fig. 3D). Both ischemic heart disease (unadjusted OR 0.53 [95% CI 0.44–0.64]), cerebrovascular disease (unadjusted OR 0.32 [95% CI 0.12–0.84]) and peripheral artery disease (unadjusted OR 0.48 [95% CI 0.34—0.66]) were associated with lower mortality (Additional file 1: Fig. S1, Additional file 2: Fig. S2, Additional file 3: Fig. S3). The results were maintained significant after further adjustments in all cases (Additional file 1: Fig. S1, Additional file 2: Fig. S2, Additional file 3: Fig. S3). Finally, the overall odds ratio (OR) for previous macrovascular disease and use of IMV during hospitalization was 0.52 (95% CI 0.40–0.68) in the unadjusted analyses (Fig. 4A), with little evidence of heterogeneity (I^2^ 37%). In the multivariable analyses, the results were similar after adjusting for age and sex (model 1: OR 0.63 [95% CI 0.47–0.85]) (Fig. 4B) and age, sex, type of diabetes, arterial hypertension, and the presence of microvascular disease (model 2: OR 0.61 [95% CI 0.45–0.83]) (Fig. 4C). Ischemic heart disease (unadjusted OR 0.55 [95% CI 0.43–0.71]) was associated with lower mortality (Additional file 1: Fig. S1, Additional file 2: Fig. S2, Additional file 3: Fig. S3). The results were maintained significant after further adjustments in all cases (Additional file 1: Fig. S1, Additional file 2: Fig. S2, Additional file 3: Fig. S3). The association between cerebrovascular disease and peripheral artery disease and use of IMV during hospitalization was not tested for insufficient number of events.

## Discussion

This is the first European retrospective study to specifically investigate the association between previous macrovascular disease and severe outcomes of people with diabetes mellitus hospitalized for COVID-19. The present study demonstrates that the presence of macrovascular complications (ischemic heart disease, stroke, peripheral artery disease) was associated with higher mortality. These findings remain significant after further adjustments for age, sex, type of diabetes, arterial hypertension, microvascular disease, ethnicity, and BMI. However, the proportions admitted to ICU and treated with IMV during hospitalization were lower for patients with previous macrovascular disease, reflecting clinical decisions around ICU admission.

COVID-19 is known to be associated with poorer outcomes for those with long-term conditions such as diabetes, and several potential mechanisms have been proposed [22]. Moreover, age, sex, ethnicity, deprivation, and other comorbidities such as obesity, hypertension and cardiovascular disease contribute to the increased risk [23]. The underlying mechanisms resulting in adverse outcomes in subjects with diabetes hospitalized for COVID-19 are therefore complex and unknown. In that sense, it has been suggested that both impaired glucose regulation and hyperglycemia [24] and the visceral adipose tissue expansion (and its associated ectopic fat depots) that characterize patients with diabetes and/or obesity activate the inflammatory cascade, increasing the production of interleukin-6, which has been proposed as a determinant factor of the “cytokine storm” associated with severe forms of COVID-19 [25].

A recent meta-analysis, including 158 observational studies with a total of 270,212 participants, of whom 57,801 had diabetes, reported that people with diabetes were at higher risk of COVID-19-related mortality with an OR 1.87 (95% CI 1.61–2.17) and higher risk of ICU admission (1.59 [95% CI 1.15–2.18]) and ventilation requirements (1.44 [95% CI 1.20–1.73]) in comparison to subjects without diabetes [11]. In addition, cardiovascular disease is common comorbidity observed in patients with COVID-19, associated with increased severity and mortality [22]. In that sense, it has been reported that patients with COVID-19 who have either hypertension or cardiovascular disease have an approximately 3–fourfold higher risk of developing severe disease [26]. By contrast, recent observational studies have demonstrated a significant association between statins (with anti-inflammatory and vasculo-protective effects) and reduced mortality in patients hospitalized with COVID-19, especially those with diabetes [27].

Moreover, an adverse effect on outcomes of diabetic complications in patients with diabetes during the COVID-19 pandemic has been recently identified. In a national population-based study in Scotland, associations with fatal or critical care unit treated COVID-19 among people with diabetes adjusted for age, sex, diabetes duration, and type of diabetes were reported for 35 factors, including heart disease (OR 2.43 [95% CI 2.14–2.75]), history of hospital admission with diabetic ketoacidosis (OR 2.87 [95% CI 1.85–4.46]), microalbuminuria (OR 1.35 [95% CI 1.16–1.58]), macroalbuminuria 1.92 [95% CI 1.52–2.43]) and severe retinopathy (OR 1.92 [95% CI 1.52–2.43]) [28]. The CORONADO study found that both microvascular (OR 2.14 [95% CI:1.16–3.94]) and macrovascular (OR 2.54 [95% CI: 1.44–4.50]) complications were independently associated with the risk of death on day seven of admission after adjusting for age, sex, comorbidities/complications, and glucose-lowering and anti-hypertensive treatment [13, 14, 19]. By contrast, no association was found between mortality (death by day seven of admission) and micro- or macrovascular complications in the ACCREDIT Study [15]. Nevertheless, both cohorts differ in several aspects such as mean age (69.8 in the CORONADO study vs. 74.1 for the ACCREDIT study cohort), median BMI (28.4 kg/m^2^ vs. 27.6 kg/m^2^), the mean HbA1c (8.1% vs. 7.7%), which may partially explain the different results regarding the outcomes [13–15, 19]. Our analysis suggests that participants living with diabetes hospitalized for COVID-19 with previous macrovascular complications (ischemic heart disease, stroke, peripheral artery disease) have an approximately 50% higher risk of mortality compared to people with no history of macrovascular disease after adjusting for all available confounding factors and that ischemic heart disease and stroke are the main contributors to this higher risk. However, proportions admitted to ICU or treated with IMV during hospitalization were lower for people with previous macrovascular disease. These results suggest unmeasured differences that may explain these opposing relationships, such as (1) the severity of the disease, (2) early mortality leading to potentially fewer patients admitted to ICU or meeting intubation criteria in the group of people with macrovascular disease or (3) the criteria used for ICU admission or IMV. In addition, it should be emphasized that our analysis revealed moderate heterogeneity in mortality and ICU admission for people with diabetes and previous cardiovascular disease with differences in the strength of the relationship between cohorts. While the higher mortality among people with history of macrovascular disease was consistently higher across the four cohorts, there were more marked differences regarding ICU admission and the use of IMV, being lower compared to the Spanish cohorts in the UK (ICU admission) and French cohorts (both ICU admission and use of IMV).

Our study is limited by the heterogeneity of data collection methods across the nations due to the use of databases that were designed separately and not specifically to answer the study question and, also by missing data. The meta-analysis combines the individual datasets to increase power but masks heterogeneity across nations. The relatively large proportion of people with missing data on one or more variables in the UK and French cohorts has occurred as a consequence of using routinely collected data from clinical practice and could introduce bias if data are not missing at random. As shown in Table 1, summary measures of the distribution of most variables used in model 3 (age, sex, type of diabetes, arterial hypertension, BMI, microvascular disease, macrovascular disease, death and ICU admission) were similar or had only modest differences between people with and without missing data in both UK and French cohorts. People of non-white ethnicity were over-represented in the missing data group in the UK cohort but not in the French cohort. Clinical data of the whole-episode, such as vital signs or arterial gasometry parameters, clinical severity scores (i.e. MEWS or CURB-65 score) or markers of inflammation (PCR, IL-6, serum ferritin) were not collected, as well as other potential confounding factors. In addition, the small sample size of patients with type 1 diabetes included made it impossible to analyze both groups separately to evaluate the potential differences between both. Lastly, we focused on people hospitalized for COVID-19; thus, our results cannot be generalized to all people with diabetes and COVID-19, especially those with less severe forms of the disease.

## Conclusions

In conclusion, this large multinational study of people with diabetes mellitus hospitalized for COVID-19 demonstrates significant associations between previous macrovascular disease and higher mortality and with lower ICU admission and the use of IMV during hospitalization. This study is the first specifically designed to evaluate the association of macrovascular complications (ischemic heart disease, stroke, or peripheral artery disease) as main exposure with mortality, intensive care unit admission and mechanical ventilation in people with diabetes mellitus hospitalized for COVID-19 in Europe. Our findings highlight the importance correctly assess the prognosis and intensive monitoring in this high-risk group of patients and emphasize the need to design specific public health programs aimed to prevent SARS-CoV-2 infection in this subgroup (i.e. reinforcing vaccination campaigns). Nevertheless, further studies are required to confirm and extend these findings in these and other populations.

## Supplementary Information

**Additional file 1: Figure S1.** Odds ratio for the association between mortality (1**A**–**C**), intensive care unit admission (2**A**–**C**) and use of invasive mechanical ventilation during the hospitalization (3**A**–**C**) and ischemic heart disease in each of the four cohorts and overall. I^2^ indicates heterogeneity in the estimates. Odds ratio estimates adjusted for: model 1: age and sex (**B**); model 2: model 1 + type of diabetes, arterial hypertension, and the presence of microvascular disease (**C**).

**Additional file 2: Figure S2.** Odds ratio for the association between mortality (1**A**–**C**), intensive care unit admission (2**A**–**C**) and and stroke in each of the four cohorts and overall. I^2^ indicates heterogeneity in the estimates. Odds ratio estimates adjusted for: model 1: age and sex (**B**); model 2: model 1 + type of diabetes, arterial hypertension, and the presence of microvascular disease (**C**).

**Additional file 3: Figure S3.** Odds ratio for the association between mortality (1**A**–**C**), intensive care unit admission (2**A**–**C**) and peripheral artery disease in each of the four cohorts and overall. I^2^ indicates heterogeneity in the estimates. Odds ratio estimates adjusted for: model 1: age and sex (**B**); model 2: model 1 + type of diabetes, arterial hypertension, and the presence of microvascular disease (**C**).

## Data Availability

Data are available on request from corresponding author and/or national study leads with appropriate data governance permission.

## Abbreviations

CKD: Chronic kidney disease
COVID-19: Coronavirus disease 2019
ICU: Intensive care unit
IMV: Invasive mechanical ventilation
OR: Odds ratio
SARS-CoV-2: Severe acute respiratory syndrome coronavirus 2
SD: Standard deviation
95% CI: 95% Confidence interval
