# Supplementary figures and images for: The association between macrovascular complications and intensive care admission, invasive mechanical ventilation, and mortality in people with diabetes hospitalized for coronavirus disease-2019 (COVID-19)
Source: Cardiovasc Diabetol. 2022 Oct 19;21:216. doi: 10.1186/s12933-022-01657-8 (PMC9580453; doi:10.1186/s12933-022-01657-8)

## Slide 1
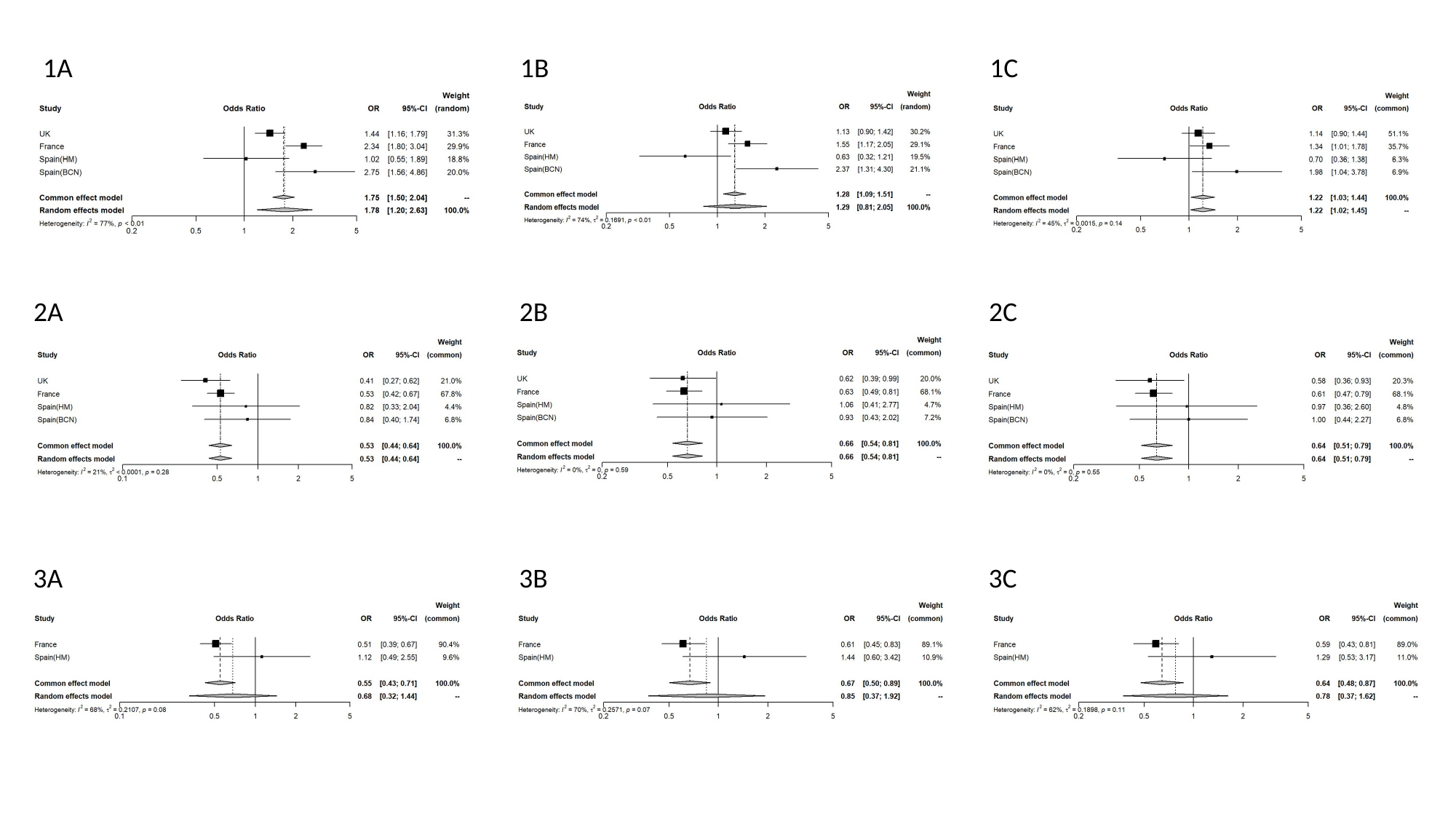

1A
1B
1C
2A
2B
2C
3A
3B
3C

Supplement: Supplementary file 1 — Additional file 1: Figure S1. Odds ratio for the association between mortality (1A–C), intensive care unit admission (2A–C) and use of invasive mechanical ventilation during the hospitalization (3A–C) and ischemic heart disease in each of the four cohorts and overall. I2 indicates heterogeneity in the estimates. Odds ratio estimates adjusted for: model 1: age and sex (B); model 2: model 1 + type of diabetes, arterial hypertension, and the presence of microvascular disease (C). [file 12933_2022_1657_MOESM1_ESM.pptx]

## Slide 1
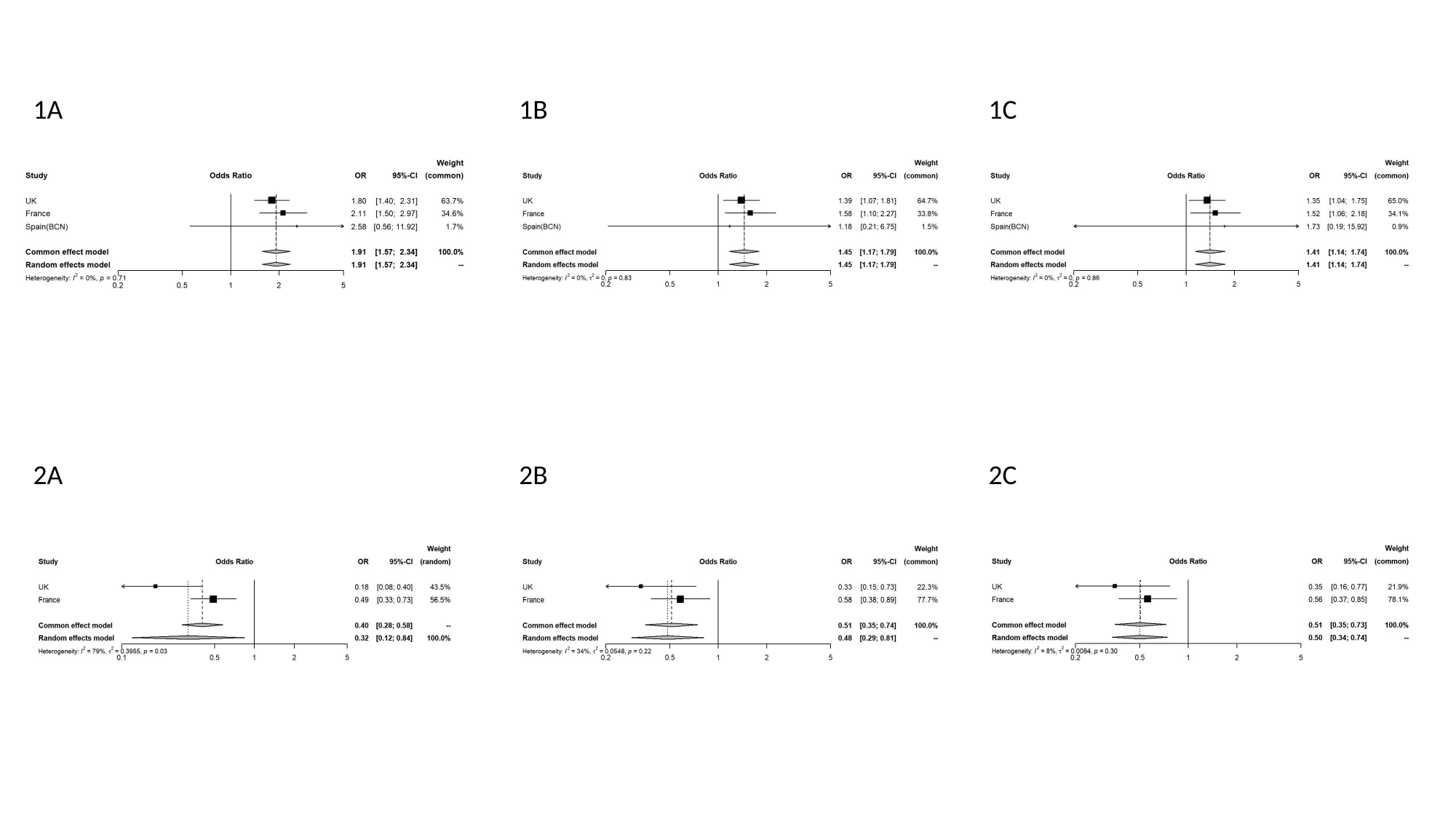

1A
1B
1C
2A
2B
2C

Supplement: Supplementary file 2 — Additional file 2: Figure S2. Odds ratio for the association between mortality (1A–C), intensive care unit admission (2A–C) and and stroke in each of the four cohorts and overall. I2 indicates heterogeneity in the estimates. Odds ratio estimates adjusted for: model 1: age and sex (B); model 2: model 1 + type of diabetes, arterial hypertension, and the presence of microvascular disease (C). [file 12933_2022_1657_MOESM2_ESM.pptx]

## Slide 1
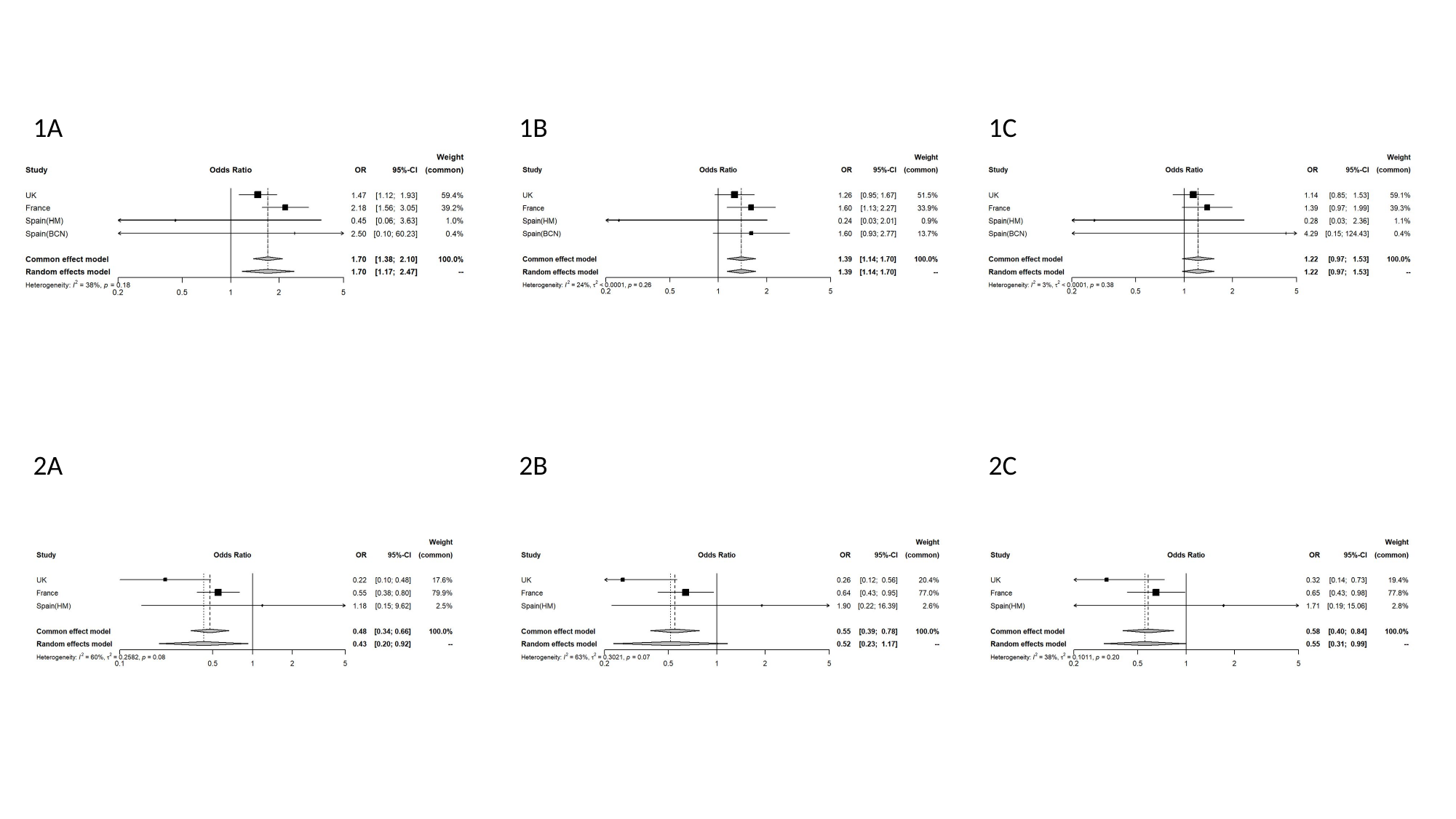

1A
1B
1C
2A
2B
2C

Supplement: Supplementary file 3 — Additional file 3: Figure S3. Odds ratio for the association between mortality (1A–C), intensive care unit admission (2A–C) and peripheral artery disease in each of the four cohorts and overall. I2 indicates heterogeneity in the estimates. Odds ratio estimates adjusted for: model 1: age and sex (B); model 2: model 1 + type of diabetes, arterial hypertension, and the presence of microvascular disease (C). [file 12933_2022_1657_MOESM3_ESM.pptx]
